# Superior sagittal sinus occlusion combined with dural arteriovenous fistula presenting as Fahr-like pattern: a case report

**DOI:** 10.3389/fsurg.2026.1700579

**Published:** 2026-02-12

**Authors:** Shang Xiang, Jingjing Wu, Shi Yang, Daiping Hua, Shiheng Shi, Han Wang

**Affiliations:** 1 Department of Neurology, The First Affiliated Hospital of Anhui University of Chinese Medicine, Hefei, China; 2 Department of Neurology, The Fifth Affiliated Hospital of Anhui University of Chinese Medicine, Lu’an, China

**Keywords:** dural arteriovenous fistula, Fahr-like pattern, intracranial calcification, involuntary movement, superior sagittal sinus occlusion

## Abstract

Dural arteriovenous fistula (DAVF) presents with diverse clinical manifestations and is susceptible to misdiagnosis. Herein, we report a case of a 64-year-old male patient who manifested involuntary tremors in the left limb, epileptic seizures, and cognitive decline. Imaging examinations revealed multiple intracranial calcifications, predominantly located in the right cerebral hemisphere, accompanied by occlusion of the superior sagittal sinus. The diagnosis of DAVF was confirmed via digital subtraction angiography (DSA). The patient's symptoms were complex, involving venous hypertension, impaired calcium metabolism, and abnormal motor cortex activity. This case emphasizes the necessity of considering DAVF in patients with unexplained movement disorders and intracranial calcium deposition, and highlights the critical role of DSA in diagnosis. By clarifying the uncommon manifestations of DAVF, this case provides a clinical reference to enhance clinicians’ awareness of this condition and underscores the importance of comprehensive multi-factor evaluation.

## Introduction

DAVF is a cerebrovascular anomaly resulting from an abnormal connection between dural arteries (or their branches) and veins (1). The precise pathogenesis of DAVF remains incompletely understood, with current research suggesting potential associations with various factors, such as trauma, surgery, tumors, hormonal imbalances, hypercoagulability, and venous sinus thrombosis (2–4). Anatomically, DAVF typically occurs in proximity to venous sinuses; common locations include the transverse-sigmoid sinus, cavernous sinus, and superior sagittal sinus (5). Notably, DAVF involving the superior sagittal sinus is relatively uncommon in clinical practice, accounting for only 4.6%–11% of reported cases according to epidemiological data (2, 6, 7). The clinical manifestations of patients with DAVF are highly diverse, encompassing a range of neurological symptoms, including pulsatile tinnitus, headache, ophthalmoplegia, chemosis, acute confusion, cognitive impairment, Parkinsonism, intracerebral hemorrhage, epileptic seizures, and neurological deficits (5, 8–10). Currently, DSA is considered the diagnostic gold standard for DAVF (11). This study presents a case of DAVF with distinctive clinical features. During the initial episode, the patient first presented with status epilepticus and hemichorea at an external medical facility. Subsequently, the disease course was complicated by various forms of involuntary movement disorders. The latest electroencephalogram revealed epileptiform discharges; however, the patient showed a limited response to conventional antiepileptic therapy. Notably, in contrast to the typical vascular calcification reported in previous DAVF literature, extensive calcified lesions were detected on the patient's cranial CT scan. Its imaging findings resembled those of Fahr syndrome (12). This unique radiographic finding may provide novel insights for DAVF diagnosis.

## Case presentations

A 64-year-old male patient presented to the emergency department of our hospital on July 30, 2025, with sudden involuntary shaking of the left limb that had persisted for 4 h. He received diazepam sedation in the emergency department and was subsequently transferred to the Department of Neurology for further management.

Previously, on July 7, 2024, the patient had been hospitalized for a 3-day episode of coma accompanied by left limb convulsions and was diagnosed with status epilepticus. Treatment included phenobarbital, levetiracetam, and diazepam for symptomatic control, followed by maintenance therapy with levetiracetam 1.0 g twice daily (bid) for seizure management. In September 2024, when left upper limb hyperactivity and involuntary shaking recurred, a consultation at an external medical facility suggested a provisional diagnosis of “hemichorea,” leading to the addition of gabapentin 0.3 g three times daily (tid) for symptom relief. The patient self-discontinued the medication after his symptoms improved.

The patient's medical history included cerebral infarction over a decade prior, for which he had received secondary preventive treatment. Additionally, he had a history of hypertension and atrial fibrillation for more than 1 year, though he was not taking any medications at the time of admission. He reported no family history of similar neurologic disorders and no history of toxic exposure.

On physical examination, the patient exhibited stable vital signs, clear mental status, coherent speech, and symmetrical pupillary responses bilaterally. Neurological examination revealed normal muscle strength and tone in all limbs; involuntary shaking was observed in the left limb, while voluntary movements of the right limb were intact. Cognitive assessment indicated mild deficits in orientation, numeracy, speech articulation, and reasoning, with a Mini-Mental State Examination (MMSE) score of 14.

Laboratory tests—including routine blood tests, C-reactive protein (CRP), urine and stool analyses, coagulation profile, immune function panel, blood glucose levels, electrolytes, renal function, thyroid function, HIV serology, liver function tests, hepatitis serological markers and parathyroid hormone (PTH) screening—were all within normal limits. Serum calcium and serum phosphorus were within normal limits, excluding secondary causes of intracranial calcification.

**Figure 1:**
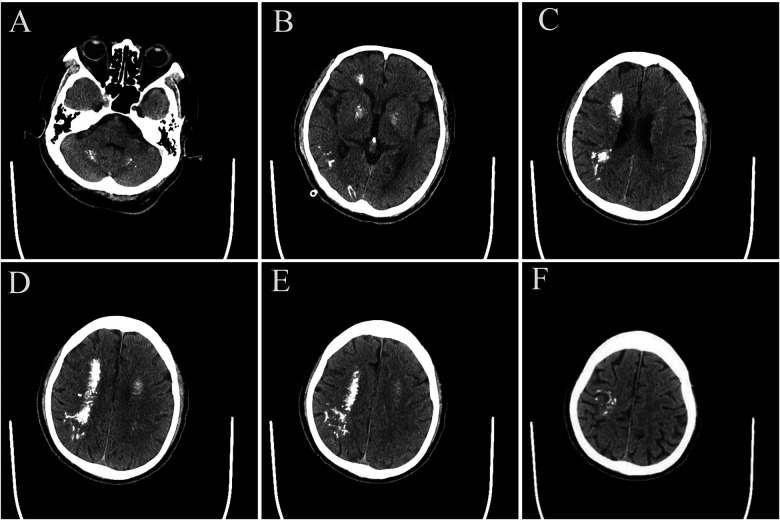
**(A–F)** cranial CT showed multiple calcified foci in the dentate nucleus region of both cerebellar hemispheres, the basal ganglia, the corona radiata region, and the right brain parenchyma, with the right cerebral hemisphere being the most prominent.

Imaging studies performed on July 30, 2025, revealed multiple calcifications in the dentate nuclei of the cerebellar hemispheres, the basal ganglia, the corona radiata, and the right cerebral parenchyma, suggestive of Fahr syndrome (Figure 1). Electroencephalogram (EEG) findings showed abnormal slow-wave activity, reduced alpha-wave activity, and a single sharp spike wave in the right central region.

**Figure 2:**
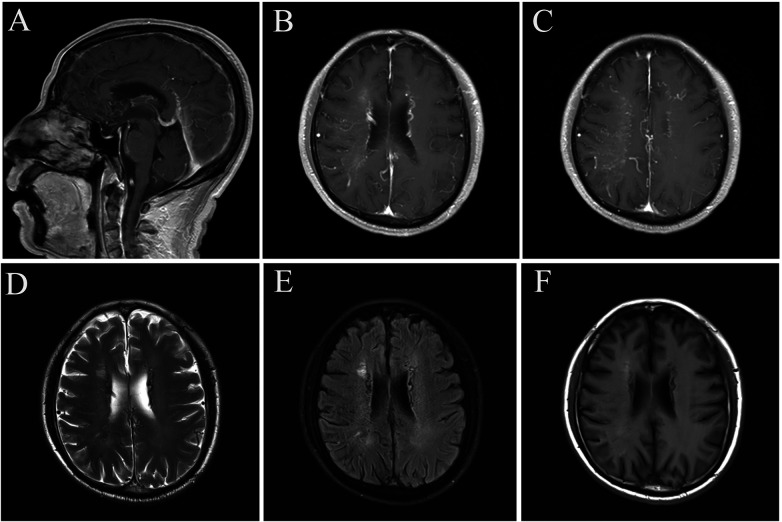
**(A–F)** cranial MRI imaging revealed multiple intricate and tortuous vascular signals within the brain, notably in the right occipital lobe and bilateral paraventricular regions. The signal within the superior sagittal sinus appeared non-uniform, and there was slight dilation of the major cerebral veins. Additionally, adjacent to the anterior horn of the right lateral ventricle, there was a patchy area displaying slightly shortened T1 and prolonged T2/FLAIR signals, with a mild enhancement effect.

Concurrently, brain magnetic resonance imaging (MRI) with contrast was performed, revealing numerous small tortuous vascular signals—particularly prominent in the right occipital lobe and bilateral ventricles. Signal intensity variability was observed in the superior sagittal sinus, while visualization of the inferior sagittal sinus and straight sinus was inadequate. Additionally, mild dilation of the great cerebral vein was detected, with no abnormal contrast enhancement foci identified in other areas of the brain parenchyma (Figure 2).

**Figure 3:**
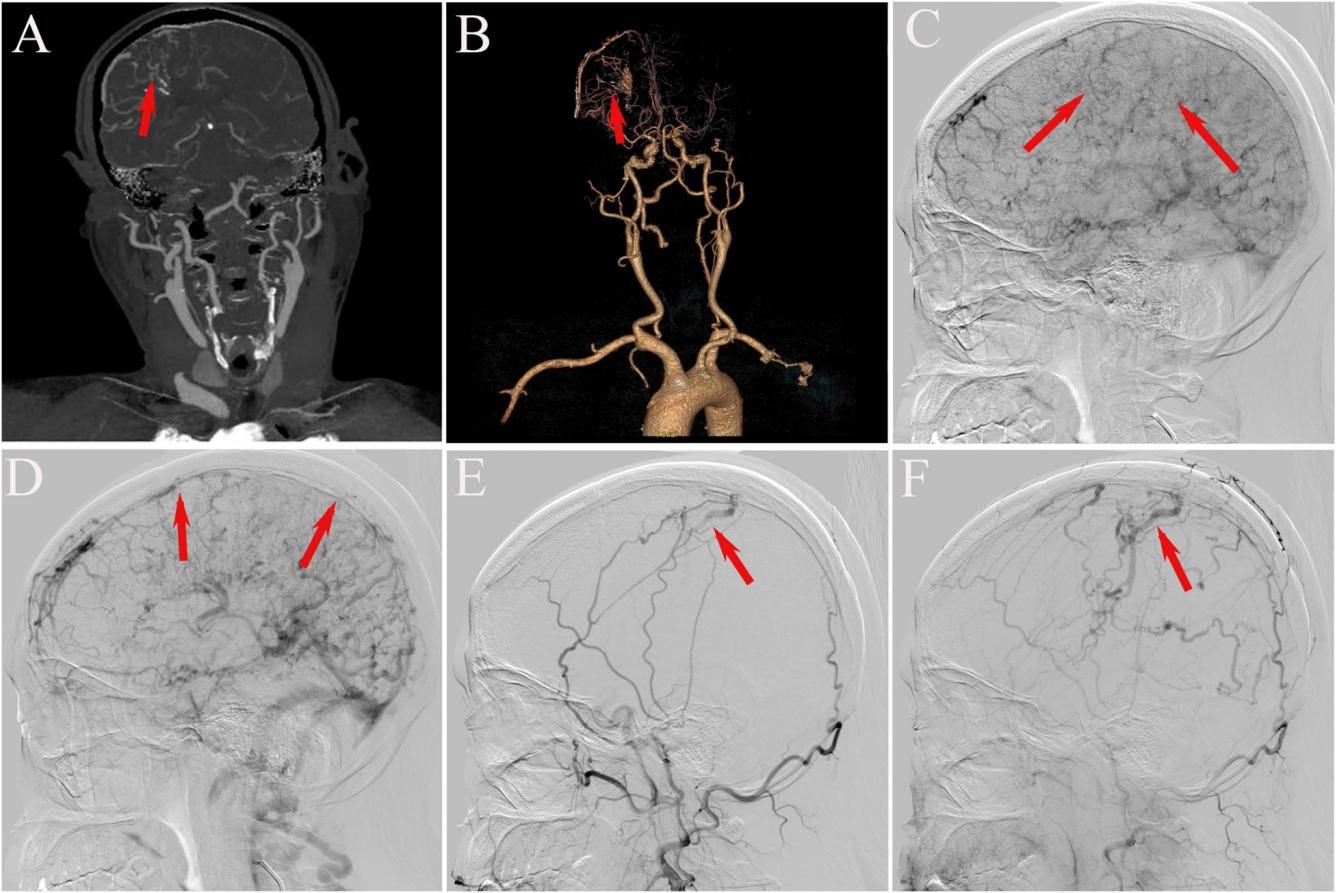
**(A,B)** head and neck vascular CTA revealed numerous slender and tortuous vascular structures within the bilateral brain parenchyma, with a predominance on the right side, as well as dilated vascular structures beneath the right cranial plate (red arrow). **(C)** Imaging of the right internal carotid artery revealed a dilated and disorganized right cerebral venous system during the venous phase (red arrow). **(D)** The absence of the superior sagittal sinus in the venous sinus phase indicated occlusion of this sinus (red arrow). **(E,F)** Bilateral external carotid arteriograms demonstrated abnormal dural vasculature draining through the cortical veins of the right parietal lobe, with an abnormally dilated draining vein in the cranium (red arrow).

**Figure 4:**
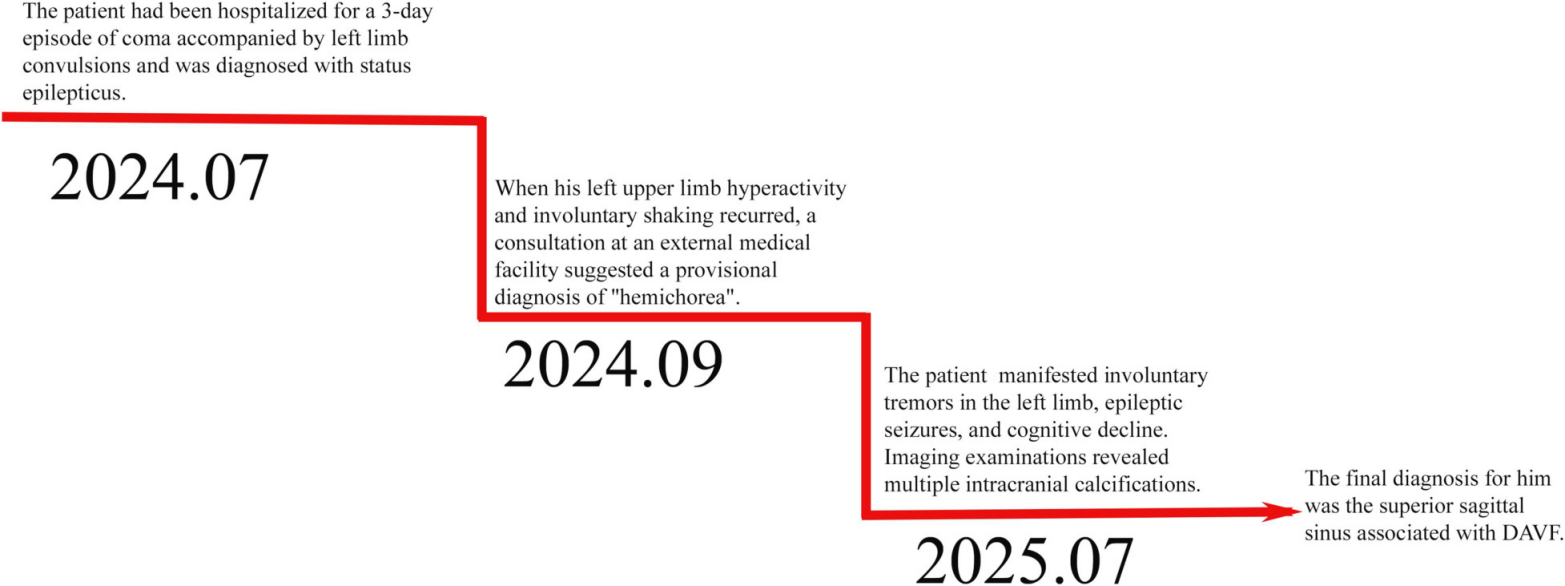
Patient clinical course timeline.

Further evaluation with head and neck computed tomography angiography (CTA) revealed atherosclerosis and moderate stenosis in the M2 segment of the left middle cerebral artery. Numerous tortuous vascular shadows were evident bilaterally within the brain parenchyma, with a notable prominence on the right side. Additionally, dilated vascular shadows were observed beneath the right cranial plate, appearing to converge toward the superior sagittal sinus—suggestive of a potential venous malformation (Figures 3A,B). On August 6, 2025, digital subtraction angiography (DSA) was performed, revealing occlusion of the superior sagittal sinus associated with DAVF (Figures 3C–F). Venous fistula closure was strongly recommended; however, the patient declined further surgical intervention due to personal considerations. Following discharge, the patient continued treatment with levetiracetam and clonazepam, in combination with secondary preventive medications for cerebral infarction. Notably, at the one-month follow-up, the patient exhibited significant improvement in limb tremor symptoms; no seizures were observed, and the patient achieved basic self-care ability. Long-term follow-up will be continued to monitor the patient's condition. The patient's disease progression and diagnostic timeline are presented in (Figure 4).

## Discussion

DAVF is an uncommon cerebrovascular anomaly characterized by abnormal direct communication between dural arteries and the venous system. Herein, we present a case of a patient with DAVF who exhibited involuntary limb movements alongside multiple intracranial calcifications. Following hospital admission, neuropsychological assessments revealed cognitive impairment; clinical and imaging findings were indicative of a Fahr-like pattern, culminating in the diagnosis of DAVF with concomitant superior sagittal sinus occlusion—confirmed by DSA. The patient's clinical phenotype and imaging characteristics implicated various pathophysiological mechanisms, including venous hypertension, aberrant calcium metabolism, seizures, and movement disorders, underscoring their clinical and scientific significance.

While the precise etiology of DAVF remains incompletely elucidated, venous sinus embolism in conjunction with venous hypertension is considered a pivotal factor (13, 14). Elevated D-dimer levels observed during the initial and subsequent episodes, coupled with pulmonary embolism, suggest a systemic hypercoagulable state in the patient—potentially predisposing them to venous sinus thrombosis and consequent DAVF formation. Venous hypertension is recognized as the fundamental pathophysiological underpinning of DAVF, a notion supported by the patient's clinical presentation in this case. DSA imaging revealed superior sagittal sinus occlusion, postulated to result from prolonged venous reflux obstruction or thrombosis. Elevated venous sinus pressure may trigger dilation of physiological dural arteriovenous connections, thereby fostering the development of pathological arteriovenous shunting—a hallmark of DAVF (1). Furthermore, venous hypertension may promote DAVF formation by stimulating neovascularization through the upregulation of pro-angiogenic factors such as vascular endothelial growth factor (VEGF) (15, 16).

The patient exhibited bilateral calcifications in the basal ganglia and cerebellar dentate nucleus with symmetrical distribution. Further laboratory investigations were performed, and all results were within normal limits, effectively ruling out common secondary causes of calcification such as hypoparathyroidism, chronic kidney disease, liver disease, diabetes mellitus, infection, inflammation, and autoimmune diseases. Although he did not have a relevant family history, primary familial brain calcification (PFBC) could not be excluded due to the patient's refusal of genetic testing. Therefore, we hypothesize that the baseline bilateral symmetric calcifications may represent age-related calcification, subclinical primary familial brain calcification, or other potential primary calcification disorders. However, asymmetrical and more pronounced calcifications were observed in the right lateral ventricle and subcortex. DAVF can cause abnormal venous return, leading to venous hypertension that impairs local cerebral perfusion and induces ischemia. Calcification in DAVF is attributed to chronic impairment of venous return, which promotes calcification deposition due to prolonged blood flow insufficiency (17). Chronic ischemia or venous stasis associated with DAVF may cause endothelial cell damage, accelerating vascular wall calcification, a condition termed “abnormal vascular calcification.” Compensatory changes in brain tissue, alterations in local blood flow, and ischemia may collectively contribute to the formation of calcification (18–21). The calcification foci observed in this case likely result from a multifactorial interplay between Fahr syndrome-like changes and DAVF-related venous stasis, leading to localized cerebral tissue hypoxia, blood-brain barrier disruption, and disturbances in calcium metabolism—all of which foster calcium deposition in the vascular wall and brain parenchyma.

Previously, the patient had been diagnosed with “status epilepticus” at another medical facility and presented with lateralized chorea during an outpatient visit. Upon admission, an EEG revealed sharp spikes in the right central region, indicating potential involvement of the motor cortex in focal motor seizures. Epilepsy is common in patients with DAVF; focal seizures are often linked to brain regions affected by the lesion, localized hypoxia from abnormal blood flow, blood-brain barrier disruption due to venous hypertension, and ischemia resulting from vasogenic and cytotoxic edema (22–24). Intracranial calcifications can disrupt localized neuronal electrical activity, leading to abnormal discharges that manifest as involuntary movements or convulsions. Despite adjustments to antiepileptic drug dosages, the patient's symptom control showed limited improvement during previous treatment; involuntary movement symptoms exacerbated markedly following discontinuation of antiepileptic drugs. Upon hospital admission, the patient's involuntary movements resolved with antiepileptic therapy and symptomatic management. The patient's fluctuating symptoms and response to antiepileptic drugs suggest a correlation between involuntary movements and seizures. The presence of extensive calcified foci in the right cerebral hemisphere, particularly in the basal ganglia region, may have directly precipitated involuntary movements by disrupting extrapyramidal pathways (25). Thus, the etiology of the patient's involuntary movements likely involves a multifactorial interplay of factors, highlighting the unique nature of this case. A limitation of this case is the absence of genetic testing and vascular intervention, which were not pursued due to the patient's personal circumstances. Long-term follow-up will be conducted to further evaluate the clinical course and outcomes.

## Conclusion

This case is of significant clinical importance, as there is a paucity of clinical reports describing DAVF presenting with manifestations consistent with a Fahr-like pattern. The patient exhibited symptoms including involuntary movements, epilepsy, and cognitive decline, and presented with intracranial calcifications. The complex causal relationships among DAVF, venous hypertension, calcified foci, epilepsy, and movement disorders require comprehensive analysis, which likely involves the interplay of venous hypertension, metabolic abnormalities, and motor circuit impairment. Patients with unexplained movement disorders and imaging evidence of intracranial calcifications should undergo thorough evaluation of vascular malformations and genetic testing, with DSA remaining the preferred diagnostic modality for complex cerebrovascular lesions. Future research should further investigate the potential causal relationship between intracranial calcification and DAVF, clarify the role of this association in the development of movement disorders, and determine the optimal therapeutic strategies for affected patients.

## Patient perspective

The patient's brother says “My brother had a loss of consciousness when he was in Shanghai last year, and he was treated in the local hospital for consideration of epilepsy, and he still had shaking of his left upper limb after taking medication, in addition to that, I found that he gradually had slow reaction and memory loss, and this time the hospitalized doctor clearly diagnosed dural arteriovenous fistula, and the condition was more complicated, and surgery was recommended, and considering the fact that my brother did not have wife and children, the family is relatively poor, so we choose to continue medication.”

## Data Availability

The original contributions presented in the study are included in the article/Supplementary Material, further inquiries can be directed to the corresponding author.
